# GWAS Fine-Mapping for Soybean First Pod Insertion Height Identifies Candidate Genomic Regions Involved in Plant Development

**DOI:** 10.3390/genes17090994

**Published:** 2026-08-24

**Authors:** Irina V. Zorkoltseva, Anatoly V. Kirichenko, Dmitriy A. Potapov, Sergey V. Kiryukhin, Sergey O. Gurinovich, Veronika I. Panarina, Revmira I. Polyudina, Elena A. Salina, Gulnara R. Svishcheva

**Affiliations:** 1 Institute of Cytology and Genetics, Siberian Branch of the Russian Academy of Sciences (SB RAS), Lavrentiev Av. 10, Novosibirsk 630090, Russia; zor@bionet.nsc.ru (I.V.Z.); kianvl@bionet.nsc.ru (A.V.K.); gulsvi@mail.ru (G.R.S.); 2 Siberian Federal Scientific Centre of Agro-BioTechnologies, Russian Academy of Sciences (RAS), Tsentralnaya St. 2b, Krasnoobsk, Novosibirsk 630501, Russia; d_potapov@ngs.ru (D.A.P.); polyudina@ngs.ru (R.I.P.); 3 Federal State Budgetary Scientific Institution (FSBSI), Federal Scientific Center of Legumes and Groat Crops, Molodozhnaya St. 10, Orel 302502, Russia; sergsv2010@mail.ru (S.V.K.); sergur17@mail.ru (S.O.G.); ver1183@yandex.ru (V.I.P.); 4 Kurchatov Genomics Center, Institute of Cytology and Genetics, Siberian Branch of the Russian Academy of Sciences (SB RAS), Lavrentiev Av. 10, Novosibirsk 630090, Russia; 5 Vavilov Institute of General Genetics, Russian Academy of Sciences (RAS), Gubkin St. 3, Moscow 119991, Russia

**Keywords:** soybean, *Glycine max*, first pod insertion height (FPIH), GWAS, fine-mapping, SuSiE, cytokinin, developmental plasticity, candidate genes, plant development

## Abstract

**Background/Objectives:** Soybean first pod insertion height (FPIH) is a key trait established during plant development, but its genetic architecture in Eurasian germplasm remains largely unknown. **Methods:** We performed a GWAS and fine-mapping for FPIH using 180 Eurasian varieties (SoySNP50K array, imputed to ~4M SNPs) phenotyped in four Russian environments (2021–2022), using two models: a residual-based and a covariate-adjusted. SuSiE fine-mapping was applied to refine candidate loci. **Results:** The covariate-adjusted model demonstrated better control of genomic inflation (λ = 1.08 vs. 1.16) and higher SNP heritability (0.381 vs. 0.014); the lower heritability in the residual-based model was expected, as this model removes environmental main effects and the genetic variance associated with them. Thus, the models are complementary: one captures stable genetic effects, the other highlights environment-dependent signals. SuSiE refined three loci. On chromosome 13, two stable independent signals (Gm13_30553403, Gm13_30909346; PIP ≥ 0.99) were identified near *MYB83* and *BEN1*, consistent across both models. On chromosome 4, the signal shifted to Gm04_36933515 (PIP = 0.9999) near *COBL4/IRX6*, although model dependency was observed, and this locus is not currently recommended for marker development. On chromosome 5, the original GWAS SNP was not causal; two tightly linked SNPs (Gm05_38427309 and Gm05_38710046, r^2^ = 0.714, PIP ≥ 0.99) formed a haplotype block located near *ARR1/ARR2* and a B-box/CCT domain gene. Crucially, this signal was absent in the residual-based model (max PIP = 0.33), suggesting that the chromosome 5 locus may modulate developmental plasticity rather than exerting a direct main effect on FPIH. However, as we did not perform formal G × E testing, we present this as a hypothesis requiring further validation. The environment-dependent behavior of this locus indicates that it should be used with caution in breeding programs and validated under specific target environments. **Conclusions:** Chromosome 13 SNPs provide stable genetic associations across environments, whereas the chromosome 5 block requires environment-specific validation. This work provides the first fine-mapped GWAS for FPIH in Russian soybean germplasm and highlights how model choice can uncover or obscure environment-dependent genetic loci affecting plant development.

## 1. Introduction

Soybean (*Glycine max* L.) is one of the most important leguminous crops worldwide, serving as a major source of vegetable oil and protein for human and animal consumption. In Russia, soybean cultivation has expanded significantly in recent decades, including in regions with challenging climatic conditions, such as Western Siberia (Novosibirsk region, ~55° N latitude). Among the agronomic traits that are established during plant development and determine the efficiency of mechanical harvesting, first pod insertion height (FPIH) is of particular importance, as it directly reflects the developmental patterning of the stem and the spatial distribution of pods. Low FPIH leads to the loss of lower pods during combine harvesting, reducing yield and economic returns. Conversely, excessively high FPIH may indicate a reduction in the total number of pods per plant. Therefore, optimizing FPIH is a key goal of soybean breeding programs.

FPIH is a quantitative trait controlled by multiple genes and influenced by environmental factors [1,2,3]. Genome-wide association studies (GWAS) have become a powerful approach for dissecting the genetic architecture of complex traits in soybean, particularly when combined with high-throughput genotyping platforms [4]. However, most GWAS have been performed on non-Russian germplasm, and the genetic architecture of FPIH in Russian soybean varieties remains largely unexplored.

Previously, we characterized the population structure and genetic diversity of 180 Eurasian soybean accessions and performed a GWAS for seed protein and oil contents using imputed genotypes [5]. In the present study, we applied the same methodological framework to identify genetic loci associated with FPIH using a collection of 180 Eurasian soybean varieties evaluated in four environments (two regions × two years). Our specific objectives were to: (1) perform a GWAS for FPIH using imputed genotype data of 180 Eurasian soybean varieties; (2) refine the localization of potentially causal variants via fine-mapping with the Sum of Single Effects (SuSiE) method [6,7]; (3) identify stable SNP associations across different environmental conditions; and (4) propose candidate genes underlying the detected loci as hypotheses for future functional validation. Thus, dissecting the genetic architecture of FPIH contributes to our understanding of plant developmental processes.

## 2. Materials and Methods

### 2.1. Soybean Population

The study analyzed a dataset comprising 180 soybean (*G. max*) accessions, including 87 Russian and European cultivars and 93 breeding lines from Western Siberia. Detailed information on the accessions, their origins, and pedigree is provided in our previous publications [5,8]. Eleven cultivars were kindly provided by the Federal Scientific Center of Legumes and Groat Crops (FSC LGC, Orel, Russia). The majority of the accessions have been collected and maintained by the Siberian Federal Research Center of Agro-BioTechnologies of the Russian Academy of Sciences (SFSCA RAS, Novosibirsk, Russia).

### 2.2. Phenotype Data for FPIH

FPIH was measured on plants grown in two geographically distinct regions of Russia: the Novosibirsk region (Western Siberia, continental climate, 55° N) and the Orel region (European part of Russia, temperate continental climate, 53° N). Plants were cultivated during two consecutive growing seasons (2021 and 2022). Details of the agronomic practices and field experiment designs have been described previously by [8].

FPIH was defined as the distance (in cm) from the soil surface to the insertion point of the first pod on the main stem. Measurements were made in triplicate for each accession in each environment, and the mean values were used for subsequent analysis. The final dataset included four “region and year” combinations (Nsk 2021, Nsk 2022, Orel 2021, Orel 2022). The phenotypic data are presented in Supplementary Table S1.

### 2.3. Genotype Data and Imputation

Genomic DNA was extracted from 3–4-day-old seedlings using the CTAB method [9]. Genotyping was performed using the SoySNP50K iSelect BeadChip array [10,11] at the Genomic Centre of the Institute of Cytology and Genetics SB RAS (Novosibirsk, Russia). Raw data were processed with GenomeStudio v.2 software (Illumina Inc., San Diego, CA, USA) and converted to PLINK format. The reference genome Wm82.a1 was obtained from SoyBase [12] (https://www.soybase.org/tools/snp50k/, accessed on 30 September 2024).

Imputation was performed in two steps following our previously described protocol [5]. In the first step, our genotypic data were imputed using the SoySNP50K genotypes of the USDA Soybean Germplasm Collection (13,545 samples, 39,551 SNPs after quality control) from SoyBase (https://www.soybase.org/, accessed on 16 August 2026) using BEAGLE v.5.4 software [13,14] with optimal parameters for soybean (ne = 10, window = 200, err = 0.0005, impsegment = 30) [15]. In the second step, the imputed data were further imputed using 302 resequenced soybean accessions [16] with the same BEAGLE parameters.

After imputation, SNPs were filtered by imputation quality score (r^2^ > 0.7) and minor allele frequency (MAF > 0.01), resulting in 3,997,265 high-quality SNPs for subsequent GWAS.

### 2.4. Phenotypic Data Analysis

Descriptive statistics (mean, standard deviation, coefficient of variation) were calculated for each of the four unique environments (Nsk 2021, Nsk 2022, Orel 2021, Orel 2022). Next, we reformatted the phenotypic data into a long format to combine all environments into a single dataset. This allowed us to use a linear mixed model (LMM) to partition the variance in FPIH between genetic and environmental sources. LMM was specified as:

*y* ~ Region × Year + (1|ID),

where *y* is the vector of FPIH values; ID represents individual soybean accessions (total *N* = 180). In this model, Region × Year denotes the main fixed effects of Region, Year, and their interaction. The term (1|ID) is a random intercept that accounts for the fact that the same soybean genotypes were measured in multiple environments (repeated measurements). Only non-missing observations were included in the phenotypic analysis and subsequent GWAS. The final dataset comprised *n* = 706 (= 179 + 170 + 178 + 179) phenotypic records from the four environments combined (see Table 1 for the exact *N* per environment).

All statistical analyses were performed in R version 4.5.1 [17]. Data manipulation was carried out using the tidyverse package [18], and LMM was fitted using the lmerTest package [19]. Normality of the model residuals was checked using the Shapiro–Wilk test. The LMM implemented in GEMMA assumes normality of the residuals, not of the raw phenotype. Given the large sample size (*n* = 706) and the high Shapiro–Wilk statistic (*W* = 0.987), we considered the residuals sufficiently normal for the analysis. We did not apply a rank-based inverse normal transformation, as the residuals met the assumptions of the model and such transformations can sometimes introduce artifacts in genetic association studies.

### 2.5. Genome-Wide Association Analysis

GWAS was performed using the linear mixed model (LMM) implemented in GEMMA (Genome-wide Efficient Mixed Model Association) software v.0.98.5 [20]. To integrate data from the four “region and year” combinations and increase statistical power, we applied the genotypic twins (synthetic samples) approach [5]. In essence, each original sample was duplicated three times, resulting in a fourfold increase in the effective sample size. The original sample and its three duplicates were treated as observations sharing identical genotypes (“genotypic twins”) but having different trait values corresponding to different environments.

Two distinct GWAS models were fitted:

**Model 1**: **Covariate-adjusted model** (environment and SNP tested simultaneously). Environmental factors (region, year, and their interaction) were included directly as fixed-effect covariates within the LMM. The dependent variable was the original FPIH value. This model estimates the SNP effect while jointly accounting for environmental variation. The model can be written as:

$$y=\mu +X_{SNP}\cdot \beta_{SNP}+X_{Region}\cdot \beta_{Region}+X_{Year}\cdot \beta_{Year}+X_{Region\times Year}\cdot \beta_{Region\times Year}+u+\varepsilon ,$$

where *y* is the vector of original FPIH values (all 706 non-missing observations); *μ* is the overall intercept; *X_SNP_* is the vector of SNP genotypes (0, 1, or 2 copies of the minor allele); *β_SNP_* is the fixed effect of the SNP being tested; *X_Region_*, *X_Year_*, and *X_Region×Year_* are design matrices for the fixed effects of region, year, and their interaction; *β_Region_*, *β_Year_*, and *β_Region×Year_* are the corresponding fixed-effect coefficients; *u* is the random polygenic effect, where $u\;\sim \;N\left(0,\;R\cdot \sigma_{g}^{2}\right)$ with *R* being the genomic relationship matrix (GRM); ε is the residual error, $\varepsilon \;\sim \;N\left(0,\;I\cdot \sigma_{e}^{2}\right)$ with *I* being the identity matrix.

**Model 2: Residual-based model** (two-step model: first environmental factors are tested, then SNP). The original FPIH value was first adjusted for environmental effects by regressing out the fixed effects of region and year. The residuals from this regression were then used as the dependent variable in the LMM, with only the intercept as a fixed effect. This approach removes environmental variation prior to association testing. Specifically, residuals were obtained in two steps. First, a reduced LMM including only the environmental fixed effects and the random polygenic effect (no SNP term) was fitted:

$$y=\mu +X_{Region}\cdot \beta_{Region}+X_{Year}\cdot \beta_{Year}+X_{Region\times Year}\cdot \beta_{Region\times Year}+u+\varepsilon$$

Residuals were then calculated as *r = y* − *ŷ*, where *ŷ* is the predicted value from the fixed environmental effects only; critically, the random accession effect u was not subtracted at this stage, so the residuals retain genetic variance. Second, these residuals were used as the dependent variable in an LMM testing only the SNP effect: $r=\mu +X_{SNP}\cdot \beta_{SNP}+u+\varepsilon$, where *u* is the same random polygenic effect, and *ε* is the residual error. Including *u* in both steps ensures that population structure and relatedness are properly accounted for throughout. The relative contribution of environmental factors to FPIH was assessed separately using phenotypic mixed models (see Section 3.2).

The genomic relationship matrix (GRM) was calculated according to VanRaden’s method 2 [21] using the R function calc_gnrm() (https://github.com/soloboan/GNRM_genomicpedigree/, accessed on 16 August 2026). For GRM calculation, SNPs with MAF > 0.05 were used. For association testing, GEMMA applied an internal MAF filter of 0.01. The GRM was calculated on the 180 unique genotypes only (180 × 180 matrix) and then expanded to 720 × 720 by replicating rows and columns to match the expanded phenotypic dataset; the duplicate rows were not treated as independent samples for kinship estimation, but rather ensure that the four environmental records for each genotype share identical kinship coefficients. The same GRM was used in both Model 1 and Model 2, so that any differences between the models are attributable to the treatment of environmental covariates rather than to differences in the kinship matrix or REML estimates.

The Wald test was used to calculate *p*-values. The genome-wide significance threshold was set at *p* < 0.05/33,171 = 1.5 × 10^−6^, where 33,171 is the number of SNPs before imputation. We used the pre-imputation marker count rather than the total number of imputed SNPs (~4 million) for the Bonferroni correction because imputation increases marker density but does not create new independent genetic information; the effective number of independent tests in soybean, given its LD structure, is much closer to the number of tag SNPs on the array (~30,000–40,000) than to the imputed marker count [22,23]. This approach is consistent with previous GWAS studies in soybean using imputed data [5]. The suggestive threshold was set at *p* < 1.5 × 10^−5^. The inflation factor (λ) was calculated as the median of the observed χ^2^ test statistic divided by the expected median. For the residual-based model (Model 2), which showed moderate genomic inflation (λ = 1.16), we additionally applied post hoc genomic control correction to the z-statistics: corrected z-statistics were calculated as $z_{corrected}=z_{observed}/\sqrt{\lambda }$, and corrected *p*-values were then derived from the standard normal distribution. This correction was applied only to Model 2, to assess which associations remained significant after accounting for inflation.

Manhattan and quantile–quantile (QQ) plots were generated using the R library qqman (v.0.1.8; [24]).

### 2.6. Fine-Mapping with SuSiE

To refine the localization of potentially causal variants, we performed fine-mapping using the SuSiE method implemented in the R package susieR (v.0.14.2) [6,7]. SuSiE decomposes the genetic signal into a sum of single effects, estimating multiple potentially causal variants simultaneously and computing posterior inclusion probability (PIP) for each SNP. Fine-mapping was performed using the susie_rss() function, which employs z-statistics and an LD correlation matrix: z-statistics were obtained from the GEMMA GWAS output; LD matrices were calculated for each region of interest using LDstore v2.0 [25] based on imputed genotypes (MAF > 0.01). LD matrix calculations were performed in PLINK v.1.9 [26] using the --ld flag, within a 3000 kb window (i.e., ±1500 kb around each lead SNP) for loci showing significant/suggestive GWAS associations. The sample size was *n* = 706, corresponding to the total number of non-missing phenotypic observations across all environments (179 + 170 + 178 + 179), derived from the genotypic twins approach. Default priors (prior_variance = 50, estimate_residual_variance = FALSE) were used to avoid overfitting, as recommended for moderate sample sizes.

Convergence was defined as an ELBO change < 1 × 10^−5^ over 10 iterations. For each 95% credible set, we calculated the minimum pairwise correlation among included SNPs (purity); sets with purity < 0.5 were considered unreliable [7]. All reported credible sets had purity > 0.8, except for the spurious L = 3 sets (purity < 0.3), which were excluded. The following parameters were used for SuSiE fine-mapping: the maximum expected number of potentially causal signals per region was set to L = 2 (primary analysis) and L = 3 (stability assessment); confidence level for credible sets was 0.95; and a SNP was considered potentially causal if its posterior inclusion probability (PIP) was ≥0.95.

SuSiE performs a form of conditional analysis by jointly modelling all genetic effects within a region. Unlike stepwise conditional analysis, which requires iterative conditioning on the top SNP, SuSiE inherently accounts for LD structure and determines the minimum number of independent signals. This allows us to distinguish genuine independent signals from spurious associations driven by LD.

### 2.7. Candidate Gene Identification (Post GWAS)

For each fine-mapped potentially causal SNP, we identified the nearest gene using the SoyBase genome annotation platform (https://legacy.soybase.org/dlpages/#genomesequence, accessed on 16 August 2026). Functional annotations were retrieved using the SoyBase Gene Annotation Tool (https://legacy.soybase.org/genomeannotation/, accessed on 16 August 2026).

## 3. Results

### 3.1. Phenotypic Variation of FPIH

**Figure 1:**
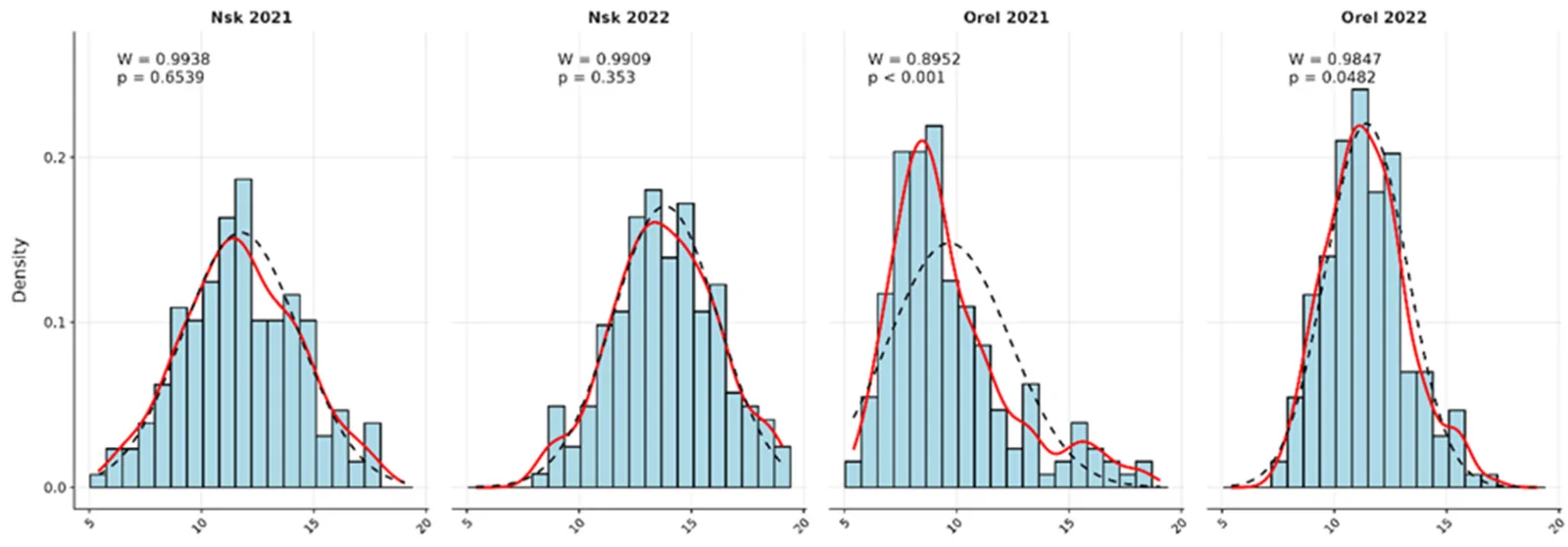
Frequency distributions of the original FPIH values. Blue histograms: observed original distributions. Red solid lines: kernel density estimates. Black dashed lines: theoretical normal distributions. W: Shapiro–Wilk test statistic. *p*: significance level (*p* < 0.001 indicates non-normality). Each panel represents a different experimental environment (region and year): Nsk (Novosibirsk) 2021, Nsk 2022, Orel 2021, or Orel 2022.

The phenotypic distributions of FPIH across the four environments are summarized in Table 1 and visualized in Figure 1. In both years, FPIH values were higher in Novosibirsk than in Orel, and were higher in 2022 than in 2021 in both regions. The coefficient of variation (CV) decreased from 2021 to 2022 in both regions, indicating reduced relative variability under more favorable growing conditions. The most pronounced deviation from normality was observed in Orel 2021 (*W* = 0.895, *p* < 0.001), likely reflecting greater environmental heterogeneity in that particular field season.

The LMM was fitted to 706 observations from the four environments combined, reflecting the total number of non-missing phenotypic records (see Table 1 and Supplementary Table S1 for details). The LMM test revealed that the Region × Year interaction was not significant (*p* > 0.05), whereas the main effects of Region and Year were highly significant (*p* < 0.001). This indicates that the environmental main effects were consistent across regions, although this does not directly address genetic stability across environments.

**Figure 2:**
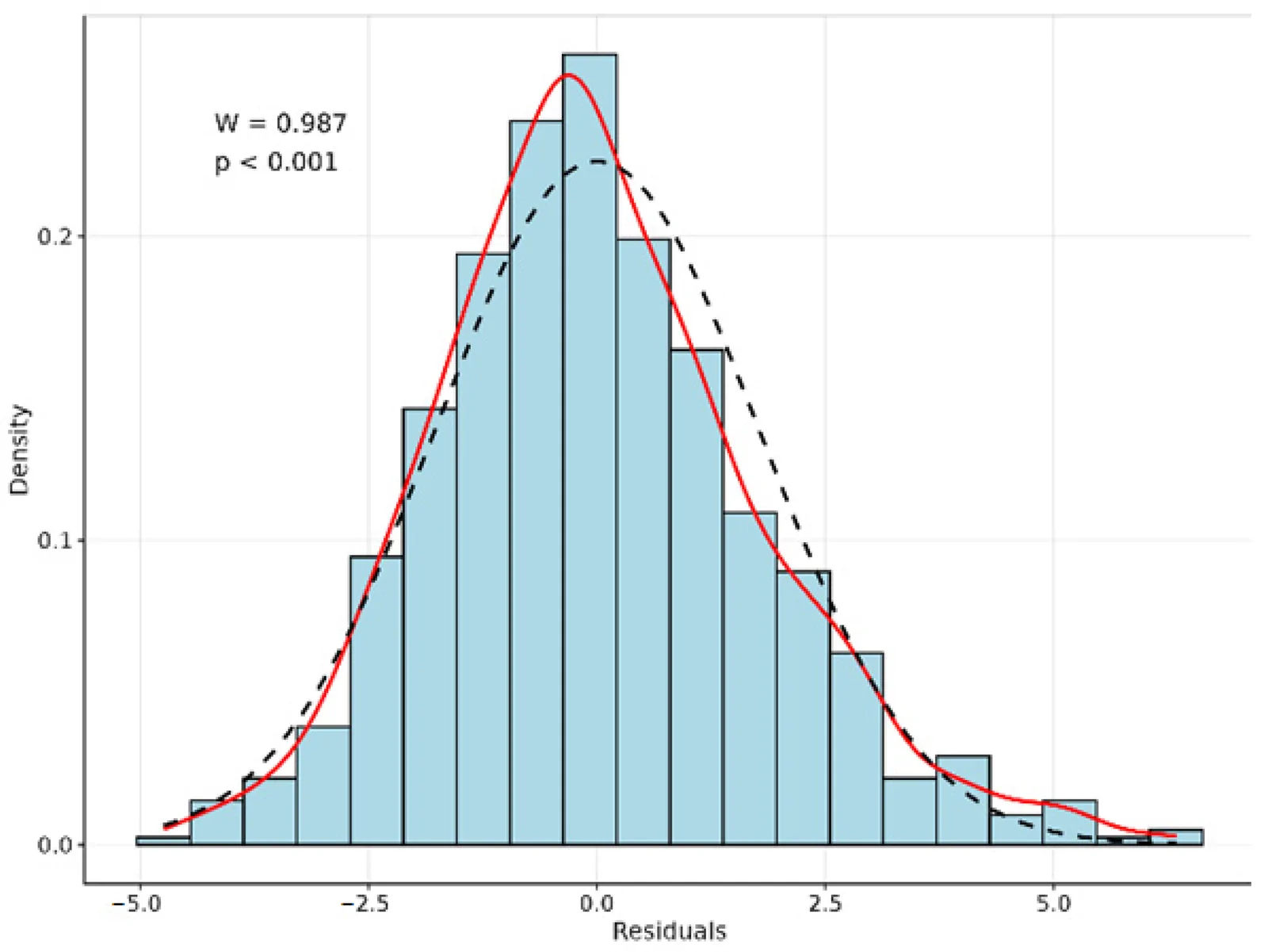
Frequency distribution of the LMM residuals from FPIH values. Blue histogram: observed residual distribution. Red solid lines: kernel density estimate. Black dashed line: theoretical normal distribution. W: Shapiro–Wilk test statistic. *p*: significance level (*p* < 0.001 indicates non-normality).

The Shapiro–Wilk test detected a significant deviation from normality in the LMM residuals (*p* < 0.05; Figure 2); however, the high *W* statistic (0.987) and large sample size (*n* = 706) suggest that the LMM is robust to this minor violation, making the residuals suitable for GWAS.

### 3.2. GWAS Results

**Figure 3:**
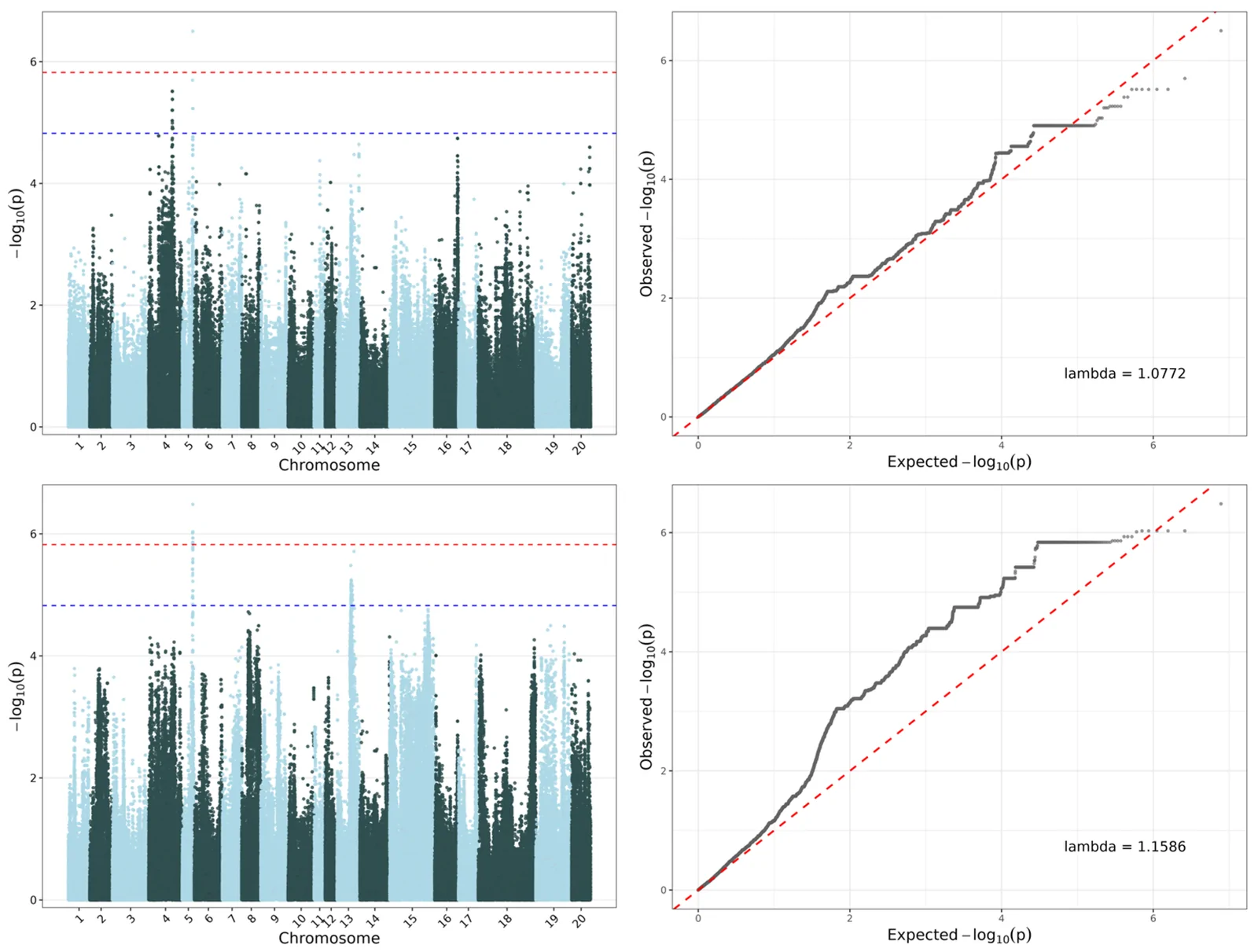
Manhattan and QQ plots for the covariate-adjusted model ((**top**), Model 1) and the residual-based model ((**bottom**), Model 2). The plots show the −log_10_ transformed *p*-values. In the Manhattan plots (**left**), the horizontal red line indicates the significance level (1.5 × 10^−6^), and the blue line indicates the suggestive level (1.5 × 10^−5^). In the QQ plots (**right**), the red dashed line indicates the expected distribution of *p*-values under the null hypothesis (no signals), while the black solid line shows the observed distribution.

GWAS was performed using the genotypic twins approach (see Section 2.5 for details). In this approach, each original sample was duplicated three times, such that each genotype had four rows corresponding to the four environments (Nsk 2021, Nsk 2022, Orel 2021, Orel 2022; Nsk = Novosibirsk), with each row containing the same genotype information but different phenotypic values, integrating phenotypic data from all four “region and year” combinations. Two models were tested: Model 1, a covariate-adjusted model (environment as fixed covariate), and Model 2, a residual-based model (trait adjusted for environment). Manhattan and QQ plots are presented in Figure 3.

#### 3.2.1. Model 1

The covariate-adjusted model showed good genomic control, with an inflation factor λ = 1.08, indicating that population stratification was adequately accounted for (Figure 3, top panel). SNP heritability for FPIH was estimated at 0.381 (SE = 0.045), reflecting a moderate to high genetic component.

Model 1 identified one genome-wide significant SNP on chromosome 5: glyma.Wm82.gnm1.Gm05_38952569 (Wm82.a1: 38,952,569) (Table 2). In addition, one suggestive SNP was identified on chromosome 4 (glyma.Wm82.gnm1.Gm04_36878574). Although this SNP did not reach the genome-wide significance threshold, we carried the chromosome 4 locus forward to fine-mapping for three reasons: (1) it was the only suggestive locus on a chromosome other than 5 and 13; (2) it was consistently detected in both models; and (3) the region contains candidate genes (*COBL4*/*IRX6* (involved in cellulose biogenesis and secondary cell wall formation)) with known functions in cell wall biogenesis, which are biologically relevant to stem development and pod height. We acknowledge, however, that fine-mapping a sub-threshold signal is unusual, and results from this locus should be interpreted with particular caution. No other genomic regions reached the suggestive threshold in this model.

**Table 2:** Summary of loci identified in both models.

Chr	SNP	Position (Wm82.a1)	A1	A0	MAF	Beta	SE	*p*-Value	Model
4	Gm04_36878574	36,878,574	T	G	0.152	1.007	0.214	3.07 × 10^−6^	1
5	Gm05_38952569	38,952,569	G	T	0.160	1.060	0.205	3.15 × 10^−7^	1
5	Gm05_38952569	38,952,569	G	T	0.160	0.462	0.089	3.29 × 10^−7^	2
13	Gm13_30553403	30,553,403	C	T	0.343	0.337	0.070	1.94 × 10^−6^	2

Note: A1—minor allele; A0—major allele; MAF—minor allele frequency; SE—standard error; Model 1—covariate-adjusted model; Model 2—residual-based model. Loci with *p* < 1.5 × 10^−6^ are considered genome-wide significant; loci with *p* < 1.5 × 10^−5^ are considered suggestive. The fine-mapped potentially causal SNPs corresponding to these loci are presented in Table 3; note that the fine-mapped SNPs differ from the original GWAS SNPs in some cases due to signal shifting after accounting for LD.

**Table 3:** Final Potentially causal SNPs Selected for Candidate Gene Analysis.

Chr	Potentially Causal SNP	Position (Wm82.a1)	Distance from Original GWAS SNP	PIP (Exact)	*p*-Value
13	Gm13_30553403	30,553,403	0 (original)	0.9992	1.94 × 10^−6^
13	Gm13_30909346	30,909,346	~356 kb	0.9978	5.29 × 10^−4^
4	Gm04_36933515	36,933,515	~55 kb	0.9999	4.14 × 10^−6^
5	Gm05_38427309	38,427,309	~525 kb	0.9997	2.00 × 10^−6^
5	Gm05_38710046	38,710,046	~242 kb	0.9912	3.49 × 10^−5^

Note: PIP, posterior inclusion probability; ~, approximately. PIPs were calculated for Model 1 (covariate-adjusted model) with L = 2 (primary analysis). These values are statistical estimates and require experimental validation.

#### 3.2.2. Model 2

The residual-based model, which attempts to remove environmental effects prior to GWAS, showed moderate inflation (λ = 1.16) (Figure 3, bottom panel). The SNP heritability dropped dramatically to 0.014, indicating a substantial loss of genetic signal.

This model detected over 100 SNPs exceeding the genome-wide significance threshold. These SNPs formed a single extended haplotype block of ~620 kb on chromosome 5, rather than a sharp peak. After correction of z-statistics for genomic inflation, only SNP glyma.Wm82.gnm1.Gm05_38952569 retained significance, suggesting that most associations in this model were likely false positives driven by environmental confounding. This correction was performed using standard genomic control: for each SNP, the corrected z-statistic was calculated as $z_{corrected}=z_{observed}/\sqrt{\lambda }$ (λ = 1.16 for Model 2), and corrected *p*-values were derived from the standard normal distribution (see Section 2.5).

The residual-based model identified one suggestive SNP on chromosome 13 (glyma.Wm82.gnm1.Gm13_30553403) that was not detected in the covariate-adjusted model at the suggestive threshold.

The key results from both models are summarized in Table 2.

### 3.3. Fine-Mapping Results

We note that the fine-mapping results presented here are based on the same panel used for the initial GWAS, which can lead to inflated PIPs and does not guarantee reproducibility. Therefore, all fine-mapped SNPs should be considered as hypotheses requiring independent validation. Throughout this section, we refer to SNPs with PIP ≥ 0.99 as potential candidate variants within the statistical framework of SuSiE, rather than as confirmed causal variants.

Fine-mapping with SuSiE was performed for each model across the three regions identified in the GWAS: one genome-wide significant locus on chromosome 5 and two suggestive loci on chromosomes 4 and 13. For each of the three regions (chromosomes 4, 5, and 13; Table 2), windows of 3000 kb (±1500 kb around each lead SNP) were analyzed. The primary analysis used L = 2 (maximum expected number of potentially causal signals), with L = 3 used for stability assessment.

#### 3.3.1. Chromosome 13

The suggestive SNP glyma.Wm82.gnm1.Gm13_30553403 was identified as a candidate causal variant in both models with PIP ≥ 0.99.

For the covariate-adjusted model, SuSiE identified two independent signals with PIP ≥ 0.99: the original SNP (Gm13_30553403) and a second SNP (Gm13_30909346) located ~356 kb away. With L = 2, both signals were stable. Increasing L to 3 produced an additional credible set of 202 SNPs with very low PIP (<0.005), which was considered an overfitting spurious signal due to insufficient sample size for L = 3. This determination was based on two pre-specified criteria: (1) the total posterior mass of the additional credible set was below 0.95; and (2) the average PIP within the set was very low (mean PIP = 0.0047), consistent with the recommendation that credible sets containing many low-PIP SNPs are unlikely to represent genuine signals [6].

For the residual-based model, SuSiE also confirmed the original SNP (PIP = 0.9992) and identified the same second signal (Gm13_30909346, PIP = 0.9978). With L = 3, two additional SNPs (Gm13_29142930 and Gm13_29237095) appeared with PIP = 0.999, but these had unusually low z-statistics and high *p*-values, suggesting they may be false positives. Therefore, the L = 2 result was retained as the most stable, yielding two independent potentially causal variants on chromosome 13, consistent across both model specifications.

#### 3.3.2. Chromosome 4

The original GWAS SNP Gm04_36878574 was not causal in either model.

For the covariate-adjusted model, SuSiE identified SNP Gm04_36933515 with PIP = 0.9999, located ~55 kb from the original SNP. Only one independent signal was found (stable at L = 2 and 3). This is a classic example of signal shifting due to LD.

For the residual-based model, SuSiE identified a different SNP: Gm04_35479141 (PIP = 0.995), located ~1.4 Mb from the original SNP, as a candidate causal variant. With L = 3, an additional SNP (Gm04_35801172) appeared but had a very low z-statistic (0.947) and high *p*-value (0.348), indicating it is spurious. The discrepancy between the two models in the localization of the potentially causal SNP on chromosome 4 (Gm04_36933515 in Model 1 vs. Gm04_35479141 in Model 2, separated by ~1.4 Mb) is a significant result that undermines confidence in either candidate. Given this inconsistency, we do not currently recommend this locus for marker development; additional studies with independent populations and functional validation are required before this locus can be considered for breeding applications.

#### 3.3.3. Chromosome 5

The original significant SNP Gm05_38952569 was not causal (PIP = 0.093 at L = 1). SuSiE identified two SNPs with PIP > 0.99: Gm05_38427309 (~525 kb from the original) and Gm05_38710046 (~242 kb from the original). Both are located proximal to the original signal. The distance between the two potentially causal SNPs is ~283 kb. LD analysis between these two SNPs revealed r^2^ = 0.714, indicating that they are in strong LD and do not represent independent potentially causal signals. Therefore, despite both receiving PIP > 0.99, they likely tag a single potentially causal variant or a single haplotype block rather than two independent effects. With L = 2, both SNPs appeared stable; with L = 3, Gm05_38427309 retained a PIP of 0.93, while Gm05_38710046 dropped to 0.12, further supporting Gm05_38427309 as the primary tag SNP. This pattern is consistent with a single underlying potentially causal variant captured by two highly correlated markers.

Critically, in Model 2, the signal on chromosome 5 completely disappeared (max PIP = 0.33). This indicates that the association of chromosome 5 variants with FPIH is entirely explained by environmental covariates (region, year, and their interaction). In other words, this locus may control developmental plasticity rather than exerting a direct main effect on FPIH. However, formal G × E testing was not performed due to limited statistical power, and the candidate genes at this locus are located 400–550 kb from the fine-mapped SNPs; these limitations should be borne in mind when interpreting this result.

#### 3.3.4. Selection of Potentially Causal SNPs for Candidate Gene Analysis

Based on stability across models and biological interpretability, the following SNPs (PIP ≥ 0.99 from Model 1 with L = 2) were selected for candidate gene analysis (Table 3).

### 3.4. Candidate Genes

Given the extended LD on chromosome 5 (620 kb haplotype block in the residual-based model), a 500 kb window was used for candidate gene identification on this chromosome. For chromosomes 4 and 13, a standard 200 kb window was applied. Only genes with direct functional relevance to plant height, cell wall, or hormone signaling are shown (Table 4).

On chromosome 13, the candidate SNPs are located very close to the candidate genes: Gm13_30553403 is approximately 2 kb from *MYB83* (a regulator of secondary cell wall development), and Gm13_30909346 is approximately 1 kb from *BEN1* (involved in brassinosteroid metabolism), increasing confidence in these genes as plausible candidates. On chromosome 5, by contrast, the candidate SNPs are located at considerable distances from the nearest annotated genes with known functions in plant architecture—approximately 400 kb from *ARR1*/*ARR2* (type-B cytokinin regulators) and approximately 549 kb from the B-box/CCT domain gene (*CONSTANS*-like transcription factors that mediate photoperiodic responses)—and, given the extended LD in this region (620 kb), these genes should be regarded as regional candidates rather than confirmed causal genes.

Note on chromosome 5 candidate genes: The nearest annotated genes with known functions in plant architecture are located 395–400 kb (*ARR1*/*ARR2*) and 549 kb (B-box/CCT) from the potentially causal SNPs. The extended LD in this region (620 kb haplotype block) may permit long-range regulatory interactions. Alternatively, the potentially causal variants may reside in non-coding regulatory elements (e.g., enhancers or long non-coding RNAs) that influence expression of distant genes. Experimental validation is required to establish the functional target.

## 4. Discussion

### 4.1. Environmental Stability and Heritability of FPIH

Before discussing our findings in detail, we highlight three caveats central to their interpretation: (1) fine-mapping was performed on the same panel used for the initial GWAS, which can inflate posterior inclusion probabilities and does not guarantee reproducibility in an independent population; (2) we did not perform formal genotype-by-environment (G × E) interaction testing, so the proposed role of the chromosome 5 locus in developmental plasticity remains a hypothesis derived from model comparison rather than a statistically confirmed interaction; and (3) the candidate genes proposed for the chromosome 5 locus are located 400–550 kb from the fine-mapped SNPs, well beyond the distance typically used to assign positional candidacy with confidence. These caveats are revisited throughout the Discussion and detailed further in Section 4.7.

FPIH is significantly influenced by both region and year, but the non-significant Region × Year interaction suggests stable genetic control across environments. This agrees with the moderate-to-high SNP heritability (0.381) we estimated, which falls within the broad range (0.00–0.74) reported for FPIH in soybean [3]. Higher FPIH in Novosibirsk than in Orel may reflect longer day length and lower temperatures during the growing season in Western Siberia, which affect stem elongation. The increase in FPIH from 2021 to 2022 in both regions likely reflects more favorable weather conditions in 2022, as also evidenced by the reduced coefficient of variation.

### 4.2. Comparison of Two GWAS Models

A key methodological advantage of this study is the use of imputed genotypes, which substantially increases marker density and enables fine-mapping of potentially causal SNPs. Imputation is therefore not merely a supplementary technique but a critical step for precise localization of variants underlying quantitative traits, such as FPIH.

Beyond imputation, the choice of GWAS model proved equally important. The covariate-adjusted model, which included region, year, and their interaction as fixed-effect covariates, was superior to the residual-based model. It improved genomic control (λ = 1.08 vs. 1.16) and increased the SNP heritability from 1.4% to 38.1%. However, the lower heritability in Model 2 is partly expected, as this model removes environmental main effects before GWAS and, consequently, also removes any genetic variance that operates through environmental modulation. The comparison between models therefore reflects not only statistical performance but also the different genetic components that each model is designed to target.

The residual-based model can mask critical types of signals: direct SNP effects that correlate with environmental factors, G × E interactions, and loci controlling developmental plasticity. The residual-based model, which attempts to remove environmental effects prior to GWAS, resulted in a dramatic loss of genetic signal and an inflated number of false-positive associations. This highlights the importance of properly accounting for environmental covariates within the GWAS model rather than through pre-correction.

The genotypic twins approach proved effective in integrating data from multiple environments while preserving the ability to estimate environmental effects. This approach integrated phenotypic data from up to four environments per genotype (706 observations in total), thereby increasing statistical power compared to single environment analyses.

### 4.3. Contribution of Fine-Mapping to Interpreting GWAS Results

Fine-mapping with SuSiE substantially refined the localization of potentially causal variants. Traditional GWAS identified three associated regions (chromosomes 4, 5, and 13), but the exact positions of potentially causal SNPs remained uncertain due to LD. SuSiE confirmed some original signals, revealed additional independent signals, and showed that some original associations were driven by LD with true causal SNPs.

#### 4.3.1. Chromosome 13—Two Independent Signals

The original SNP Gm13_30553403 was confirmed as potentially causal (PIP = 1.00), and a second independent signal (Gm13_30909346, ~355 kb away) was identified. This suggests either two different genes/regulatory elements influencing FPIH, or a complex LD structure with two independently associated blocks. The signal persisted in the residual-based model, indicating a direct genetic effect on FPIH.

#### 4.3.2. Chromosome 4—Signal Shifting

The original SNP Gm04_36878574 was not causal. Fine-mapping shifted the signal to Gm04_36933515 (~55 kb away)—a typical case where the original GWAS SNP is in strong LD with the true causal variant but is not causal itself. As discussed above, the discrepancy between models in the localization of the causal signal on chromosome 4 undermines confidence in either candidate, and we do not currently recommend this locus for marker development.

#### 4.3.3. Chromosome 5—Environment-Dependent Signal (Developmental Plasticity)

The original significant SNP Gm05_38952569 was not causal (PIP = 0.093). Instead, two SNPs (Gm05_38427309 and Gm05_38710046 with PIP ≥ 0.99) were identified as potentially causal in the covariate-adjusted model. However, these two SNPs are in strong LD (r^2^ = 0.714). Consequently, they do not represent two independent potentially causal variants but rather a single haplotype block likely containing one potentially causal variant (or a set of tightly linked variants). The nearest functionally relevant genes are *ARR1*/*ARR2* (cytokinin signaling) located approximately 400 kb away.

The signal on chromosome 5 disappeared in the residual-based model (max PIP = 0.33). An alternative interpretation of the chromosome 5 fine-mapping results is that the signal disappeared in Model 2 simply because of reduced statistical power, given the drop in heritability from 0.381 to 0.014. Under this scenario, the locus could still have a direct main effect on FPIH, but Model 2 lacked the power to resolve it. We consider this less likely for two reasons: first, the original GWAS SNP remained significant in Model 2 (*p* = 3.29 × 10^−7^), indicating that power for detecting some signal was retained; second, the failure of SuSiE to identify a credible causal variant despite an adequate sample size and clear LD structure suggests that the signal is not a simple, unitary effect. Nevertheless, formal G × E testing is required to distinguish between these competing hypotheses, and we therefore present the developmental-plasticity interpretation as the more biologically plausible, but not definitively proven, explanation. This interpretation is consistent with the presence of a B-box/CCT domain gene near these SNPs (~549 kb), which is known to mediate photoperiodic responses in plants [27].

We did not conduct a statistical test for G × E (genotype by environment) on chromosome 5 due to limited power with 180 accessions. Nevertheless, the disappearance of the association signal in the residual-based model, combined with the presence of candidate genes involved in photoperiodic and cytokinin pathways, suggests an environment-dependent effect requiring larger panels. This finding has important implications for breeding: unlike the stable locus on chromosome 13, the chromosome 5 locus should be used with caution and validated under specific target environments. It may be particularly valuable for developing region-specific varieties adapted to Western Siberia versus Central Russia.

### 4.4. Biological Interpretation of Potentially Causal SNPs

Chromosome 13—stable effect: The two potentially causal SNPs are in a region containing genes related to cell wall development and hormonal regulation, including *MYB83* (secondary cell wall regulator) and *BEN1* (brassinosteroid metabolism). The stability of this signal across environmental adjustments suggests that these genes contribute to FPIH through a direct, environment-independent mechanism. *MYB83* is a master regulator of secondary cell wall formation, and *BEN1* is involved in brassinosteroid homeostasis—both processes directly affect stem elongation and mechanical properties.

Chromosome 4—model-dependent effect: Although this locus reached only suggestive significance in the initial GWAS, the potentially causal SNP Gm04_36933515 (from the covariate-adjusted model) is near *COBL4*/*IRX6* and *COBL4* genes, involved in cellulose biogenesis and secondary cell wall formation—consistent with the role of stem mechanical properties in determining FPIH. However, the discrepancy between models warrants caution in interpreting this locus. The alternative candidate Gm04_35479141 from the residual-based model is located in a region with less clear functional annotation, further highlighting the need for experimental validation.

Chromosome 5—environment-dependent effect: The two potentially causal SNPs are in a region containing *ARR1*/*ARR2* gene (Type-B cytokinin response regulators) and a B-box/CCT domain gene potentially involved in photoperiodic regulation, at distances of 400–550 kb. However, given the extended linkage disequilibrium (LD) in this region (620 kb) and the substantial physical distance, these genes should be considered as potential candidates rather than confirmed potentially causal genes. It remains equally possible that the true causal variants reside in non-coding regulatory elements or uncharacterized genes in closer proximity. Functional validation (e.g., gene expression analysis or mutant studies) is required to establish the target genes at this locus. The complete disappearance of the signal in the residual-based model suggests that these genes may be involved in environment-dependent regulation of stem growth. Cytokinin signaling is known to be modulated by environmental cues such as light and temperature, and the B-box/CCT domain is characteristic of *CONSTANS*-like transcription factors that mediate photoperiodic responses. We hypothesize that variants in this region affect the responsiveness of stem elongation to environmental signals, rather than setting a fixed baseline height.

Recent experimental evidence supports a role for cytokinin in regulating basal internode elongation and pod height in soybean. Thapar et al. [28] demonstrated that while exogenous cytokinin inhibits internode elongation (antagonizing gibberellin), genotypes with taller lowest pod height exhibit elevated levels of endogenous isopentenyladenine (IPA). This recent experimental evidence provides supportive context for, but does not confirm, our hypothesis regarding the chromosome 5 locus. This suggests a dose-dependent and tissue-specific effect, consistent with our observation that the cytokinin-related *ARR1*/*ARR2* locus on chromosome 5 is environment-dependent and may modulate phenotypic plasticity rather than a fixed main effect.

### 4.5. Comparison with Previous Studies

Several quantitative trait loci (QTLs) for FPIH have been reported on different soybean chromosomes, including chromosomes 2, 7, 16, 17, and 20 [2]. Our fine-mapped locus on chromosome 5 (around 38.4–38.7 Mb) is in a region not previously associated with FPIH in major GWAS studies. A QTL for plant height has been reported on chromosome 5 at approximately 39.0–39.8 Mb [29], which is distinct from our fine-mapped region at 38.4–38.7 Mb. Although the two loci are on the same chromosome, they do not overlap, suggesting they may represent independent genetic determinants. The stable QTL on chromosome 13 (around 30.5–30.9 Mb) has not been previously reported for FPIH to our knowledge, representing a novel finding. The absence of previously reported major FPIH QTLs (e.g., on chromosomes 6 and 19) in our population may be explained by the unique genetic composition of Russian soybean varieties. These varieties have undergone selection under specific climatic conditions (particularly in Western Siberia) and may carry different allelic variants compared to North American or Asian germplasm.

### 4.6. Priority Regions for Validation and Breeding

Based on our fine-mapping results, we propose the following priority regions for validation and potential use in breeding programs. We emphasize that all recommendations are preliminary and require independent validation before deployment in marker-assisted selection. On chromosome 5, because the two SNPs are in strong LD (r^2^ = 0.714), either can be used as a proxy for the same haplotype block. Recommended SNPs for breeding based on fine-mapping results are presented in Table 5.

Chromosome 13 SNPs represent the primary candidates for further validation and marker development (high priority). The chromosome 5 haplotype block should be used with caution and validated under specific target environments (medium priority). The chromosome 4 locus is not currently recommended for marker-assisted selection given the discrepancy between models (low priority).

Particular attention should be given to the haplotype block on chromosome 5, as its effect appears to be strongly environment-dependent. For these markers, validation on independent panels across different ecogeographic zones of Russia is required. The stable SNPs on chromosome 13 are promising for breeding programs aiming for consistent FPIH across diverse environments. We also recommend that future studies perform fine-mapping under multiple model specifications to assess the robustness of causal variant inference.

### 4.7. Limitations

As noted in the opening of the Discussion (Section 4.1), several central caveats apply to the interpretation of our results. Here we provide a comprehensive list of study limitations. This study has several limitations.

First and most importantly, although fine-mapping identified SNPs with very high PIP (≥0.99), these results are based on the same panel used for GWAS. This leads to inflated PIPs and does not guarantee reproducibility of the causal variant assignment. Therefore, all fine-mapped SNPs, regardless of their PIP values, should be considered as hypotheses requiring validation in an independent soybean population.

Second, the genotypic twins approach assumes additive environmental effects independent of genotype. While this increased power, it may not fully capture complex G × E interactions. We acknowledge this as a limitation and note that the primary value of this approach is in increasing statistical power for main effect detection.

Third, candidate genes are based on positional and functional annotation; experimental validation (expression analysis, mutants, or complementation) is essential. This is particularly important for the chromosome 5 locus, where the causal SNPs are 400–550 kb from the nearest annotated genes.

Fourth, while we observed environment-dependent behaviour of the chromosome 5 locus, we did not perform G × E testing due to limited power. The sample size of 180 genotypes provides adequate power for main effect detection but is insufficient for reliable interaction testing. Therefore, our conclusion that these SNPs “may control phenotypic plasticity” is presented as a hypothesis derived from model comparison, not as a statistically confirmed G × E.

Fifth, the fine-mapped causal SNPs on chromosome 5 are located 400–550 kb from the nearest annotated genes with plausible functions (*ARR1*/*ARR2* and B-box/CCT). While extended LD in the region (620 kb) and documented cases of long-range regulatory elements in plants provide a potential explanation, we cannot exclude that other, uncharacterized genes or non-coding RNAs in closer proximity are the true functional targets.

Sixth, the imputation accuracy depends on genetic similarity to reference panels; rare variants (MAF < 0.01) were excluded.

Seventh, the discrepancy in causal SNP localization on chromosome 4 between the two models highlights the importance of model specification in fine-mapping. Future studies should consider both approaches and validate findings experimentally.

Finally, the exact molecular mechanisms by which *ARR1*/*ARR2* and *COBL4* markers affect FPIH remain to be elucidated.

## 5. Conclusions

This study presents the first GWAS and fine-mapping for first pod insertion height in a collection of 180 Eurasian soybean varieties using imputed genotypes. By performing fine-mapping on both covariate-adjusted and residual-based models, we were able to distinguish environment-stable loci (chromosome 13) from environment-dependent QTLs (chromosome 5) and identify a model-dependent locus (chromosome 4) requiring further validation. The chromosome 13 locus represents a robust target for breeding programs aiming for consistent FPIH across diverse environments.

Fine-mapping revealed that the two high-PIP SNPs on chromosome 5 are in strong LD and thus tag a single haplotype block, whose effect is entirely environment-dependent, suggesting a role in phenotypic plasticity. Candidate genes include type-B cytokinin response regulators (*ARR1*/*ARR2*), cell wall genes (*COBL4*, *COB*), transcription factors (*MYB83*), and the brassinosteroid metabolism gene *BEN1*. The chromosome 5 locus offers opportunities for region-specific adaptation but requires careful validation.

The covariate-adjusted GWAS model with genotypic twins proved superior to residual-based approaches. The identified potentially causal SNPs provide a foundation for marker-assisted selection aimed at optimizing FPIH in Russian soybean breeding programs, with special attention to the distinct properties of each locus.

## Figures and Tables

**Table 1:** Descriptive statistics for FPIH in Novosibirsk (Nsk) and Orel regions in 2021 and 2022.

Region	Year	N	Mean (cm)	SD	CV * (%)
Nsk	2021	179	11.75	2.59	22.0
Nsk	2022	170	13.83	2.34	16.9
Orel	2021	178	9.67	2.70	27.9
Orel	2022	179	11.43	1.81	15.8

Note: * Coefficient of variation: CV = (SD/mean) × 100.

**Table 4:** Candidate genes near fine-mapped potentially causal SNPs.

Chr	Potentially Causal SNP	Nearby Gene ID	Regional Candidate Gene	Functional Annotation	Distance to Gene (kb)
5	Gm05_38427309	Glyma05g34516	*ARR2* (AT4G16110)	Type-B cytokinin response regulator	~400
5	Gm05_38427309	Glyma05g34523	*ARR1* (AT3G16857)	Type-B cytokinin response regulator	~400
5	Gm05_38710046	Glyma05g35151	*B-box*/CCT domain (AT1G25440)	Photoperiodic transcription regulator	~549
4	Gm04_36933515	Glyma04g32120	*COBL4*/*IRX6*	Secondary cell wall biogenesis	<10 (flanking)
4	Gm04_36933515	Glyma04g32130	*COBL4* (*COBRA*-Like 4 Ortholog)	Cell wall biogenesis	<10 (flanking)
13	Gm13_30553403	Glyma13g27310	*MYB83*	Secondary cell wall regulation	~2
13	Gm13_30909346	Glyma13g27390	*BEN1*	Brassinosteroid biosynthesis	~1

Note: All potentially causal SNPs have PIP ≥ 0.99.

**Table 5:** Candidate SNPs for marker-assisted selection.

Priority	SNP	Chr	LD/Independence	Recommendation
Medium	Gm05_38427309 or Gm05_38710046	5	r^2^ = 0.714	Environment specific validation required
High	Gm13_30553403, Gm13_30909346	13	Independent signals	Wide adaptability
Low	Gm04_36933515	4	–	Not currently recommended for MAS; requires independent validation

Note: G × E, genotype-by-environment interaction; LD, linkage disequilibrium; SNPs in strong LD (r^2^ = 0.714) tag a single haplotype block and are interchangeable.

## Data Availability

The original contributions presented in this study are included in the article/Supplementary Materials. Further inquiries can be directed to the corresponding author.

## Supplementary Materials

The following supporting information can be downloaded at https://www.mdpi.com/article/10.3390/genes17090994/s1, Supplementary Table S1. Phenotypic data for FPIH in the Novosibirsk (Nsk) and Orel regions in 2021 and 2022.

## References

[1] Kang B.K., Kim H.T., Choi M.S., Koo S.C., Seo J.H., Kim H.S., Shin S.O., Yun H.T., Oh I.S., Kulkarni K.P. (2017). Genetic and Environmental Variation of First Pod Height in Soybean [*Glycine max* (L.) Merr.]. Plant Breed. Biotechnol..

[2] Jiang H., Li Y., Qin H., Li Y., Qi H., Li C., Wang N., Li R., Zhao Y., Huang S. (2018). Identification of Major QTLs Associated with First Pod Height and Candidate Gene Mining in Soybean. Front. Plant Sci..

[3] Kuzbakova M., Khassanova G., Oshergina I., Ten E., Jatayev S., Yerzhebayeva R., Bulatova K., Khalbayeva S., Schramm C., Anderson P. (2022). Height to first pod: A review of genetic and breeding approaches to improve combine harvesting in legume crops. Front. Plant Sci..

[4] Bhat J.A., Yu D. (2021). High-throughput NGS-based genotyping and phenotyping: Role in genomics-assisted breeding for soybean improvement. Legume Sci..

[5] Potapova N.A., Zorkoltseva I.V., Zlobin A.S., Shcherban A.B., Fedyaeva A.V., Salina E.A., Svishcheva G.R., Aksenovich T.I., Tsepilov Y.A. (2025). Genome-Wide Association Study on Imputed Genotypes of 180 Eurasian Soybean *Glycine max* Varieties for Oil and Protein Contents in Seeds. Plants.

[6] Wang G., Sarkar A., Carbonetto P., Stephens M. (2020). A simple new approach to variable selection in regression, with application to genetic fine mapping. J. R. Stat. Soc. Ser. B (Stat. Methodol.).

[7] Zou Y., Carbonetto P., Wang G., Stephens M. (2022). Fine-mapping from summary data with the “Sum of Single Effects” model. PLoS Genet..

[8] Perfil’ev R., Shcherban A., Potapov D., Maksimenko K., Kiryukhin S., Gurinovich S., Panarina V., Polyudina R., Salina E. (2023). Impact of Allelic Variation in Maturity Genes *E1–E4* on Soybean Adaptation to Central and West Siberian Regions of Russia. Agriculture.

[9] Rogers S.O., Bendich A.J. (1985). Extraction of DNA from milligram amounts of fresh, herbarium and mummified plant tissues. Plant Mol. Biol..

[10] Song Q., Hyten D.L., Jia G., Quigley C.V., Fickus E.W., Nelson R.L., Cregan P.B. (2013). Development and evaluation of SoySNP50K, a high-density genotyping array for soybean. PLoS ONE.

[11] Song Q., Hyten D.L., Jia G., Quigley C.V., Fickus E.W., Nelson R.L., Cregan P.B. (2015). Fingerprinting Soybean Germplasm and Its Utility in Genomic Research. G3 Genes Genomes Genet..

[12] Grant D., Nelson R.T., Cannon S.B., Shoemaker R.C. (2010). SoyBase, the USDA-ARS soybean genetics and genomics database. Nucleic Acids Res..

[13] Browning B.L., Zhou Y., Browning S.R. (2018). A one-penny imputed genome from next-generation reference panels. Am. J. Hum. Genet..

[14] Browning B.L., Tian X., Zhou Y., Browning S.R. (2021). Fast two-stage phasing of large-scale sequence data. Am. J. Hum. Genet..

[15] Chen L., Yang S., Araya S., Quigley C., Taliercio E., Mian R., Specht J.E., Diers B.W., Song Q. (2022). Genotype imputation for soybean nested association mapping population to improve precision of QTL detection. Theor. Appl. Genet..

[16] Zhou Z., Jiang Y., Wang Z., Gou Z., Lyu J., Li W., Yu Y., Shu L., Zhao Y., Ma Y. (2015). Resequencing 302 wild and cultivated accessions identifies genes related to domestication and improvement in soybean. Nat. Biotechnol..

[17] R Core Team (2025). R: A Language and Environment for Statistical Computing.

[18] Wickham H., Averick M., Bryan J., Chang W., McGowan L.D.A., François R., Grolemund G., Hayes A., Henry L., Hester J. (2019). Welcome to the Tidyverse. J. Open Source Softw..

[19] Kuznetsova A., Brockhoff P.B., Christensen R.H.B. (2017). LmerTest Package: Tests in Linear Mixed Effects Models. J. Stat. Softw..

[20] Zhou X., Stephens M. (2012). Genome-wide efficient mixed-model analysis for association studies. Nat. Genet..

[21] VanRaden P.M. (2008). Efficient methods to compute genomic predictions. J. Dairy Sci..

[22] Dudbridge F., Gusnanto A. (2008). Estimation of significance thresholds for genomewide association scans. Genet. Epidemiol..

[23] Gao X., Starmer J., Martin E.R. (2008). A multiple testing correction method for genetic association studies using correlated single nucleotide polymorphisms. Genet. Epidemiol..

[24] Turner S.D. (2018). Qqman: An R package for visualizing GWAS results using Q-Q and manhattan plots. J. Open-Source Softw..

[25] Benner C., Havulinna A.S., Järvelin M.-R., Salomaa V., Ripatti S., Pirinen M. (2017). Prospects of Fine-Mapping Trait-Associated Genomic Regions by Using Summary Statistics from Genome-wide Association Studies. Am. J. Hum. Genet..

[26] Purcell S., Neale B., Todd-Brown K., Thomas L., Ferreira M.A.R., Bender D., Maller J., Sklar P., de Bakker P.I.W., Daly M.J. (2007). PLINK: A Tool Set for Whole-Genome Association and Population-Based Linkage Analyses. Am. J. Hum. Genet..

[27] Dong L., Fang C., Cheng Q., Su T., Kou K., Kong L., Zhang C., Li H., Hou Z., Zhang Y. (2021). Genetic basis and adaptation trajectory of soybean from its temperate origin to tropics. Nat. Commun..

[28] Thapar A., Tuan P.A., Kaur A., Sharma D., Ayele B.T. (2025). Insights into the Roles of Gibberellin and Cytokinin Levels in Regulating Elongation of Basal Internodes and Lowest Pod Height in Soybean. J. Plant Growth Regul..

[29] Sha D., Zhang Z., Gu Y., Zhang S., Khattak A.N., Liu Y., Ma C., Hu M., Niu J., Yu L. (2026). High-resolution quantitative trait loci mapping and pyramiding effects of candidate genes for plant height in soybean. Plant Genome.

