# Supplementary material for: GWAS Fine-Mapping for Soybean First Pod Insertion Height Identifies Candidate Genomic Regions Involved in Plant Development
Source: Genes (Basel). 2026 Aug 24;17(9):994. doi: 10.3390/genes17090994 (PMC13606300; doi:10.3390/genes17090994)
Supplement: Supplementary file 1 [file genes-17-00994-s001.zip › genes-4437241-supplementary.pdf]

Genome-Wide Association Study and Fine-Mapping for First Pod Insertion Height in 180 Eurasian Soybean Varieties Using Imputed Genotypes

Table S1. Phenotypic data for FPIH in the Novosibirsk (Nsk) and Orel regions in 2021 and 2022.

	Nsk		Orel	
ID	2021	2022	2021	2022
1	8.90	13.07	6.70	9.60
2	10.50	11.20	6.92	11.20
3	10.75	13.23	9.30	10.80
4	12.80	12.07	9.40	11.70
5	12.30	12.30	9.00	13.00
6	12.05	14.83	6.40	11.90
7	11.93	15.30	9.30	11.30
8	10.87	12.13	9.40	9.80
9	11.47	10.60	8.70	8.50
10	8.97	12.77	8.90	10.60
11	10.70	11.43	9.10	9.40
12	11.03	13.23	7.90	9.90
13	11.93	11.77	8.64	10.00
14	11.13	14.13	11.60	9.20
15	11.77	8.73	14.20	9.50
16	11.33	17.27	9.40	11.80
17	6.70	12.50	8.00	10.80
18	12.83	16.37	8.60	10.40
20	11.10	16.57	9.64	12.40
21	14.50	12.43	14.90	12.80
22	13.80	17.67	9.90	11.30
24	17.27	12.80	11.10	13.90
25	NA	NA	17.90	15.40
26	15.30	13.93	7.10	11.50
27	14.70	18.60	13.20	14.00
28	14.10	NA	11.10	15.55
29	11.70	10.70	16.60	11.30
30	16.03	18.50	8.40	13.80
31	17.47	17.60	18.50	14.11
32	15.95	14.83	8.40	15.60
33	9.77	12.47	9.30	10.50
35	11.20	11.90	13.20	12.60
36	10.43	9.70	6.00	10.20
37	9.05	12.00	7.00	11.30
38	6.35	11.40	6.30	10.30
39	6.07	11.17	6.60	9.30
40	10.94	14.43	8.45	14.00
41	13.25	12.60	15.30	13.10
42	12.97	13.17	9.18	14.30

43	9.75	13.10	10.36	10.60
44	17.57	18.47	15.40	12.60
45	16.25	16.97	10.90	12.10
46	15.30	10.86	7.80	11.70
47	14.53	13.77	8.10	12.36
48	10.15	8.90	6.90	12.90
49	10.20	11.57	7.00	9.30
50	17.87	19.05	10.60	11.50
52	9.17	12.23	7.63	12.50
53	12.07	14.50	10.70	13.20
54	15.73	13.47	12.10	12.60
55	12.03	13.63	8.40	14.70
56	10.85	16.20	9.80	11.20
57	13.77	8.80	6.40	10.42
58	8.93	14.30	8.10	9.44
59	13.45	12.67	9.55	11.40
60	10.93	14.37	8.25	9.50
61	7.83	12.17	9.00	10.60
62	14.06	14.13	NA	14.40
63	17.27	13.77	15.70	13.45
64	10.57	13.17	8.90	12.40
65	11.56	14.97	10.50	12.70
66	11.90	16.60	17.00	15.60
67	9.73	10.85	8.60	12.20
68	10.90	14.57	8.80	10.50
69	16.33	16.67	11.50	15.27
70	10.60	8.65	8.70	12.90
71	11.40	11.47	13.40	10.10
72	14.30	12.00	7.64	11.33
73	14.10	15.50	14.80	10.70
74	11.80	12.30	11.27	11.90
76	12.23	17.90	13.00	13.70
77	13.73	12.67	10.40	12.30
78	12.93	13.67	11.40	12.30
79	11.80	9.17	5.40	9.30
80	13.17	12.80	10.90	13.50
81	11.00	17.10	10.90	9.80
82	8.53	9.53	7.80	12.70
83	12.50	16.03	10.40	10.78
84	11.43	12.13	9.30	11.09
85	5.63	11.57	5.40	8.10
86	15.91	14.63	13.10	11.70
87	9.66	12.03	16.30	12.00
88	14.47	15.63	10.45	17.00
89	10.90	11.23	8.90	12.60
90	13.33	13.57	8.90	9.20

91	13.60	16.13	16.50	12.50
92	13.90	16.00	7.60	14.90
93	14.40	15.70	12.00	10.60
94	11.87	12.63	9.20	10.40
95	14.93	16.10	11.50	11.80
96	12.87	16.20	11.60	11.10
97	12.17	13.20	10.60	12.70
98	8.07	14.37	7.80	10.40
99	12.27	11.47	8.75	11.00
100	6.90	10.95	9.00	9.20
101	9.80	11.53	7.80	8.60
102	11.60	11.85	7.20	9.22
103	12.20	13.45	8.30	9.90
104	9.84	13.80	9.20	11.11
105	16.27	13.13	8.60	7.50
106	11.70	12.53	6.90	10.90
107	9.60	10.67	7.83	9.70
108	14.60	13.77	9.40	11.20
109	9.80	10.07	6.10	12.08
110	7.23	10.53	6.50	9.00
111	10.27	16.57	7.30	12.09
112	8.33	15.70	11.20	8.90
113	7.30	16.13	7.80	10.00
114	10.03	13.40	8.40	9.30
115	14.57	14.23	8.60	11.80
116	13.40	15.00	10.60	15.70
117	15.17	17.93	10.60	13.70
118	6.97	15.77	9.50	11.60
119	13.95	14.47	9.80	12.80
120	12.53	16.03	9.40	9.90
121	10.47	18.30	9.40	10.90
122	10.90	14.87	6.30	15.00
123	8.87	14.90	9.00	12.30
124	9.23	13.43	11.80	11.63
125	12.07	15.37	8.80	12.90
126	8.23	15.33	8.00	8.30
127	11.22	10.47	6.80	8.80
128	10.05	18.83	7.60	9.90
129	11.27	16.87	12.50	13.10
130	10.20	13.63	8.80	10.60
131	7.27	14.63	9.00	8.22
132	11.93	14.60	9.30	11.29
133	8.70	14.93	10.20	10.50
134	8.52	13.17	8.40	12.40
135	9.07	15.00	8.40	10.78
136	12.40	16.47	6.70	10.70

137	10.70	15.87	7.50	9.73
138	11.60	13.47	9.20	10.90
139	8.03	10.73	7.83	10.90
140	8.53	12.63	8.10	8.30
141	11.65	12.43	8.60	11.10
142	7.77	14.53	8.09	8.55
143	10.53	15.63	7.70	9.00
144	9.93	13.35	9.90	10.50
145	14.50	13.47	11.50	11.25
146	13.30	16.33	7.90	10.10
147	13.33	14.13	6.40	11.50
148	11.97	13.90	12.40	12.10
149	16.87	12.90	11.70	11.33
150	9.37	12.40	10.00	7.80
151	10.77	12.77	7.70	11.50
152	9.28	11.33	NA	16.00
153	13.73	17.70	7.70	12.00
154	11.77	15.27	9.60	12.10
155	9.23	13.80	7.90	13.00
156	12.20	8.53	8.40	11.50
157	10.87	18.70	13.20	12.80
158	9.00	12.33	10.50	12.50
159	14.30	13.00	7.90	8.90
160	13.83	11.17	9.70	9.70
161	10.43	15.37	7.20	9.50
162	16.75	14.97	13.50	13.60
163	14.10	14.73	9.00	11.60
164	13.50	13.60	7.10	10.70
165	10.83	14.30	7.40	12.00
166	6.47	14.53	7.00	10.40
167	9.77	18.50	7.90	12.70
168	9.00	16.03	6.80	10.40
169	10.17	14.63	8.20	12.00
170	8.57	13.33	6.80	11.90
171	13.13	13.67	7.80	11.90
172	12.33	15.10	8.10	9.80
173	8.80	16.10	8.30	10.60
174	12.37	15.07	8.10	10.60
175	11.27	NA	15.50	8.90
176	14.83	15.90	10.30	11.30
177	15.05	NA	18.00	14.00
178	15.03	NA	16.00	12.30
179	14.30	NA	12.70	11.09
180	13.05	NA	13.40	9.00
182	14.90	NA	15.50	NA
183	13.80	NA	8.80	13.60

184	12.83	NA	8.30	10.40
185	12.10	8.87	10.60	11.00
186	13.00	15.23	7.30	12.56
